# Physiologically Based Pharmacokinetic Model of Brain Delivery of Plasma Protein Bound Drugs

**DOI:** 10.1007/s11095-023-03484-2

**Published:** 2023-02-24

**Authors:** William M. Pardridge

**Affiliations:** UCLA, Los Angeles, CA 90095 USA

**Keywords:** albumin, alpha-1-acid glycoprotein, blood–brain barrier, imipramine, propranolol

## Abstract

**Introduction:**

A physiologically based pharmacokinetic (PBPK) model is developed that focuses on the kinetic parameters of drug association and dissociation with albumin, alpha-1 acid glycoprotein (AGP), and brain tissue proteins, as well as drug permeability at the blood–brain barrier, drug metabolism, and brain blood flow.

**Goal:**

The model evaluates the extent to which plasma protein-mediated uptake (PMU) of drugs by brain influences the concentration of free drug both within the brain capillary compartment *in vivo* and the brain compartment. The model also studies the effect of drug binding to brain tissue proteins on the concentration of free drug in brain.

**Methods:**

The steady state and non-steady state PBPK models are comprised of 11–12 variables, and 18–23 parameters, respectively. Two model drugs are analyzed: propranolol, which undergoes modest PMU from the AGP-bound pool, and imipramine, which undergoes a high degree of PMU from both the albumin-bound and AGP-bound pools in plasma.

**Results:**

The free propranolol concentration in brain is under-estimated 2- to fourfold by *in vitro* measurements of free plasma propranolol, and the free imipramine concentration in brain is under-estimated by 18- to 31-fold by *in vitro* measurements of free imipramine in plasma. The free drug concentration in brain *in vivo* is independent of drug binding to brain tissue proteins.

**Conclusions:**

*In vitro* measurement of free drug concentration in plasma under-estimates the free drug in brain *in vivo* if PMU *in vivo* from either the albumin and/or the AGP pools in plasma takes place at the BBB surface.

**Supplementary Information:**

The online version contains supplementary material available at 10.1007/s11095-023-03484-2.

## Introduction

Drug efficacy in brain is believed to be driven by the concentration of free (unbound) drug in brain (C_u,brain_) [1]. One approach to the measurement of C_u,brain_ requires the dual measurement of the ratio of total drug in brain, divided by the total drug in plasma, or K_p,brain_, in parallel with determination of the k_p,uu,brain_, which is the ratio of C_u,brain_ divided by the free (unbound) drug in plasma, or C_u,plasma_. The C_u,brain_ and C_u,plasma_ are measured *in vitro* by equilibrium dialysis of brain homogenate and plasma, respectively [2, 3].

*In vitro in vivo* extrapolation assumes the C_u,plasma_ measured *in vitro* is a reliable index of the free (bioavailable) drug *in vivo* within the brain capillary plasma compartment, i.e., that C_u,plasma,*in vivo*_ is equal to C_u,plasma,*in vitro*_. This assumption requires that drug bound to plasma proteins, such as albumin or alpha-1 acid glycoprotein (AGP), is not available for transport *in vivo*. As reviewed by Zhang *et al*. [1], work in the 1980s provided evidence for drug delivery to organs *in vivo* *via* the plasma protein bound pool, and that this work has been largely forgotten. Recently, there has been renewed interest in drug delivery to tissues *in vivo via* the plasma protein-bound pool [4–6], a process referred to as plasma protein-mediated uptake or PMU [7].

The purpose of the present work was to develop a physiologically based pharmacokinetic (PBPK) model of brain delivery of drugs that are bound in plasma by albumin and AGP. The partly flow-partly compartmental model is tested under conditions of both the steady state and non-steady state, and is based on 18 parameters governing the kinetics of drug binding to albumin and AGP in plasma, drug binding to tissue proteins in brain, drug influx and efflux across the blood–brain barrier (BBB), drug metabolism, and cerebral blood flow. The PBPK model is examined for 2 drugs, propranolol, which undergoes modest PMU by brain from the AGP-bound pool in plasma [8], and imipramine, which undergoes extensive PMU by brain from both the albumin-bound and AGP-bound pools in plasma [9].

## Methods

**Fig. 1:**
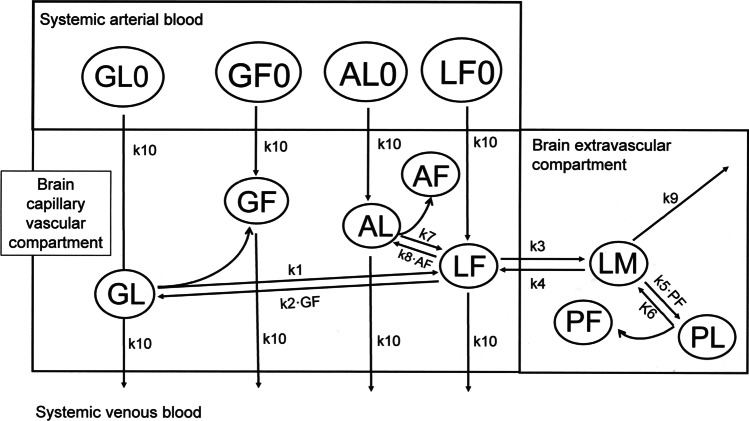
Partly flow-partly compartment PBPK model of brain delivery of drugs bound to plasma proteins such as albumin or AGP. The 11 variables, GL0, GF0, AL0, LF0, GL, GF, AL, LF, LM, PL, and PF, are distributed over 3 compartments: the systemic circulation, the brain capillary plasma volume, and the brain post-vascular volume. Within the systemic circulation, GL0, AL0, LF0, and GF0 is the concentration of globulin-bound drug, albumin-bound drug, free (unbound) drug, and free (unbound) globulin, respectively. Within the brain capillary plasma volume, GL, AL, LF, and GF is the capillary globulin-bound drug, the capillary albumin-bound drug, the capillary free (bioavailable) drug, and the capillary unoccupied globulin, respectively. Within the post-vascular brain compartment, LM, PL, and PF are the free drug in brain, the drug bound to brain tissue proteins, and the unoccupied brain tissue protein pool, respectively. The basal values for the 18 parameters of the model are defined in Table II.

The partly flow-partly compartmental model of brain drug delivery is outlined in Fig. 1. The PBPK model is comprised of 11 variables, which are also defined in Table I. The concentration of free albumin in the capillary compartment, AF, is not treated as a variable, and is approximated by the total albumin concentration, because the plasma albumin concentration is log orders higher than the total plasma drug concentration. In the non-steady state model, as in the case of oral drug administration, the total plasma drug concentration (LT0) varies with time, and LT0[t] is a 12^th^ variable. The steady state model and the non-steady state model include 18 and 23 parameters, respectively. The basal values for the model parameters for propranolol and imipramine, along with literature citations, are given in Table II. The non-steady state model was examined only for propranolol, as detailed pharmacokinetic (PK) parameters are not available following oral administration of imipramine.

**Table I:** Model Variables

Compartment	Variable	Definition
Arterial	GL0	Arterial concentration of globulin-bound drug
	AL0	Arterial concentration of albumin-bound drug
	LF0	Arterial concentration of free (unbound) drug (C_u,plasma,*in vitro*_)
	GF0	Arterial concentration of free (unbound) globulin
Brain capillary	GL	Brain capillary concentration of globulin-bound drug
	AL	Brain capillary concentration of albumin-bound drug
	LF	Brain capillary concentration of free (bioavailable) drug (C_u,plasma,*in vivo*_)
	GF	Brain capillary concentration of free (unbound) globulin
Brain	LM	Brain concentration of free (unbound) drug (C_u,brain,*in vivo*_)
	PL	Brain concentration of drug bound to cytoplasmic proteins
	PF	Brain concentration of free cytoplasmic protein

Unit of All Variables is nM. PL + PF = PT, the Total Drug Binding Tissue Protein in Brain

**Table II:** Model Parameters for Propranolol and Imipramine

Parameter	Parameter name	Propranolol (reference)	Imipramine (reference)
LT0	Total plasma drug concentration	100 nM [10]	100 nM [11]
AF	total systemic and capillary albumin concentration	800,000 nM [12]	800,000 nM [12]
GT0	total systemic globulin concentration	20,000 nM [12]	20,000 nM [12]
KA^*in vitro*^	KD of drug binding to albumin *in vitro*	290,000 nM [8]	42,000 nM [13]
KG^*in vitro*^	KD of drug binding to globulin *in vitro*	3,300 nM [8]	1,200 nM [14]
PT	total brain binding protein concentration	5,000 nM	5,000 nM
VP	brain capillary volume	0.01 L/kg [15]	0.01 L/kg [15]
VT	brain extravascular volume	0.7 L/kg [15]	0.7 L/kg [15]
k1	rate constant of drug dissociation from globulin in brain capillary	1140 min^−1^ [8]	5,400 min^−1^ [9]
k2	rate constant of drug association with globulin in brain capillary	0.06 nM^−1^ min^−1^ [16] (10^6^ M^−1^ s^−1^)	0.06 nM^−1^ min^−1^ [16] (10^6^ M^−1^ s^−1^)
k3	rate constant of drug influx from plasma to brain across BBB	66 min^−1^ [8]	150 min^−1^ [9]
k4	rate constant of drug efflux from brain to plasma across BBB	0.943 min^−1^ [8]	2.1 min^−1^ [9]
k5	rate constant of drug association with brain protein binding site	0.006 nM^−1^ min^−1^ (10^5^ M^−1^ s^−1^)	0.006 nM^−1^ min^−1^ (10^5^ M^−1^ s^−1^)
k6	rate constant of drug dissociation from brain protein binding site	0.52 min^−1^ [17]	≤ 0.5 min^−1^ [9]
k7	rate constant of drug dissociation from albumin in brain capillary	1740 min^−1^ [8]	≥ 6,000 min^−1^ [9]
k8	rate constant of drug association with albumin in brain capillary	0.006 nM^−1^ min^−1^ [18] (10^5^ M^−1^ s^−1^)	0.006 nM^−1^ min^−1^ [19] (10^5^ M^−1^ s^−1^)
k9	rate constant of drug metabolism in brain	0 min^−1^ [17]	0 min^−1^ [20]
k10	rate constant of brain capillary plasma flow	60 min^−1^ [15]	60 min^−1^ [15]
b	fractional oral bioavailability	0.3 [21]	-
s	drug dose	4,600 nmol/kg, 80 mg [22]	-
k	rate constant drug oral absorption	0.023 min^−1^ [22]	-
d	rate constant drug systemic elimination	0.0027 min^−1^ [22]	-
V	drug systemic volume of distribution	5.0 L/kg [21]	-

Plasma total drug concentration in steady state after intravenous infusion is LT0; plasma total drug concentration in non-steady state after oral administration is determined from the pharmacokinetic parameters (b, s, k, d, and V) as described in Eq. 13. KD = dissociation constant of drug binding to albumin or globulin

### Steady State Model

In the steady state model, the total plasma drug concentration, LT0, is constant as in the case of an intravenous (IV) infusion. The inputs from the systemic arterial blood to the brain capillary plasma compartment are given by k10·GL0, k10·GF0, k10·AL0, and k10·LF0 (Fig. 1). The solutions to the differential equations at steady state, where the rate of change of each variable concentration is zero, have been described previously [15], and are,

1 $$GL0=0.5KG(B-\sqrt{\left({B}^{2} -\frac{4GT0\cdot LT0}{K{G}^{2}}\right)}), where B=(1+\left(\frac{AF}{KA}\right)+\left(\frac{(GT0+LT0)}{KG}\right))$$

2 $$GF0= GT0-GL0$$

3 $$LF0= LT0/(1+\frac{AF}{KA}+\frac{GF0}{KG})$$

4 $$AL0=(\frac{AF\cdot LT0}{KA})/(1+ \frac{AF}{KA}+\frac{GF0}{KG})$$

5 $$LM=\left(\frac{VP}{VT}\right)\cdot \frac{\left[\sqrt{{\left(a2+a1\cdot b1\right)}^{2}+4\cdot a2\cdot b1\left(b2-a1\right)}-\left(a2+a1\cdot b1\right)\right]}{2\cdot a2\cdot b1}$$

5a $$a1=k10\cdot GF0-k10\cdot LF0-[\frac{k7\cdot k10\cdot ALO}{k7+k10}]$$

5b $$a2=k9+\left[k10\cdot \frac{\left(k4+k9\right)}{k3}\right]\cdot [1+\frac{k8\cdot AF}{\left(k7+k10\right)}]$$

5c $$b1=\frac{k2\cdot (k4+k9)}{k3\cdot (k1+k10)}$$

5d $$b2=k10\cdot GF0+\frac{k1\cdot k10\cdot GLO}{k1+k10}$$

6 $$GF=\frac{\left[a1+a2\cdot LM\cdot \left(\frac{VT}{VP}\right)\right]}{k10}$$

7 $$LF=\left[\frac{k4+k9}{k3}\right]\cdot \left(\frac{VT}{VP}\right)\cdot LM$$

8 $$AL=\frac{k10\cdot ALO+k8\cdot AF\cdot LF}{k7+k10}$$

9 $$GL=\frac{k10\cdot GL0+k2\cdot LF\cdot GF}{k1+k10}$$

10 $$PF=\frac{PT}{[1+\left(\frac{k5}{k6}\right)*LM]}$$

11 $$PL=PT-PF$$

12 $${K}_{p,brain}=\left(PL+LM\right)/LT0$$

The KA and KG parameters in Eqs. 1, 3, and 4 are the KA^*in vitro*^ and KG^*in vitro*^ parameters, respectively, listed in Table II.

The C_u,plasma,*in vitro*_ = LF0; the C_u,plasma,*in vivo*_ in the brain capillary = LF, and the C_u,brain,*in vivo*_ = LM. The fractional unbound drug in plasma *in vitro*, f_u,plasma,*in vitro*_ = LF0/LT0. The fractional unbound (bioavailable) drug in brain capillary plasma *in vivo*, f_u,plasma,*in vivo*_ = LF/LT0. The values for the 11 variables, for any given set of parameters, were computed with the *Solve* program of Mathematica version 12.3.1.0 (Wolfram, Champaign, IL). Equation 7 shows that, assuming drug metabolism is nil, where k9 = 0, then the LM/LF ratio, which is equivalent to the k_p,uu,brain_ [2, 3], is equal to the PS^influx^/PS^efflux^ ratio, where PS^influx^ = k3·VP and PS^efflux^ = k4·VT, and PS is the permeability-surface area product of drug transport across the BBB. These relationships predict that both LM and k_p,uu,brain_ are independent of drug binding to brain tissue proteins (PT). In contrast, the K_p,brain_, which is equal to (LM + PL)/LT0, is a function of brain tissue binding parameters, as PL is a function of PT and the k6/k5 ratio, as shown in Eqs. 10–11. The dissociation constant (KD) of drug binding to brain tissue proteins is KP and is equal to the k6/k5 ratio. The KD of drug binding to albumin *in vivo* within the brain capillary is KA^*in vivo*^ and is equal to the k7/k8 ratio. The KD of drug binding to AGP *in vivo* within the brain capillary is KG^*in vivo*^ and is equal to the k1/k2 ratio.

### Non-Steady State Model

In the non-steady state model, the total plasma drug concentration, LT0[t], is a function of time (t) after oral (PO) administration. Assuming a 1-compartment first order model of drug absorption into plasma and drug elimination from plasma [23], the LT0[t] is defined as,

13 $$LT0\left[t\right]=[{\frac{b\cdot s\cdot k}{V\cdot \left(k-d\right)}]\cdot (e}^{-d\cdot t}-{e}^{-k\cdot t})$$

where the pharmacokinetic variables are given in Table II for propranolol. The concentrations of GL0[t], GF0[t], LF0[t], and AL0[t], in the systemic arterial compartment are given by,

14 $$GL0\left[t\right]=0.5\cdot KG\cdot (B-\sqrt{\left({B}^{2} -\frac{4\cdot GT0\cdot LT0\left[t\right]}{K{G}^{2}}\right)}), where B=(1+\left(\frac{AF}{{K}_{A}}\right)+\left(\frac{GT0+LT0[t])}{KG}\right))$$

15 $$GF0[t]= GT0-GL0[t]$$

16 $$LF0[t]= LT0[t]/(1+\frac{AF}{KA}+\frac{GF0[t]}{KG})$$

17 $$AL0[t]=(\frac{AF\cdot LT0[t]}{KA})/(1+ \frac{{A}_{F}}{KA}+\frac{GF0[t]}{KG})$$

The KA and KG parameters in Eqs. 14, 16, and 17 are the KA^*in vitro*^ and KG^*in vitro*^ parameters, respectively, listed in Table II.

The rate of change of the variable concentrations in the brain capillary and the brain compartments of the PBPK model in Fig. 1 are described by the following differential equations,

18 $$\frac{dGL}{dt}={k}_{10}\cdot GL0\left[t\right]+{k}_{2}\cdot LF[t]\cdot GF[t]-{k}_{1}\cdot GL[t]-{k}_{10}\cdot GL[t]$$

19 $$\frac{dAL}{dt}={k}_{10}\cdot AL0\left[t\right]+{k}_{8}\cdot AF\cdot LF[t]-{k}_{7}\cdot AL[t]- {k}_{10}\cdot AL[t]$$

20 $$\frac{dLF}{dt}={k}_{10}\cdot LF0\left[t\right]+{k}_{1}\cdot GL\left[t\right]+{k}_{7}\cdot AL\left[t\right]+{k}_{4}\cdot LM\left[t\right]\cdot \left(\frac{VT}{VP}\right)-{k}_{2}\cdot LF\left[t\right]\cdot GF\left[t\right]-{k}_{8}\cdot AF\cdot LF[t]-{k}_{3}\cdot LF[t]-{k}_{10}\cdot LF[t]$$

21 $$\frac{dLM}{dt}={k}_{3}\cdot LF[t]\cdot \left(\frac{VP}{VT}\right)-\left({k}_{4}+{k}_{9}\right)\cdot LM[t]-{k}_{5}\cdot LM[t]\cdot PF[t]+{k}_{6}\cdot PL[t]$$

22 $$\frac{dPF}{dt}={k}_{6}\cdot PL[t]-{k}_{5}\cdot LM[t]\cdot PF[t]$$

23 $$\frac{dPL}{dt}= {k}_{5}\cdot LM[t]\cdot PF[t]-{k}_{6}\cdot PL[t]$$

24 $$\frac{dGF}{dt}={k}_{10}\cdot GF0\left[t\right]+{k}_{1}\cdot GL[t]- {k}_{2}\cdot LF[t]\cdot GF[t]-{k}_{10}\cdot GF[t]$$

The values for the model variables for propranolol (Table I), given the parameters in Table II, were solved with the *NDSolve* program of Mathematica version 12.3.1.0 (Wolfram, Champaign, IL), with the following initial conditions: LT0[0] = 0, GL0[0] = 0, LF0[0] = 0, AL0[0] = 0, GL[0] = 0, AL[0] = 0, LF[0] = 0, LM[0] = 0, PL[0] = 0, GF0[0] = GT0, GF[0] = GT0, and PF[0] = PT.

The PBPK models are based on the following assumptions. First, the volume of the brain arterial compartment is comparable to the volume of the brain capillary compartment, as demonstrated by brain imaging studies [24, 25]. Second, rates of change in the systemic arterial compartment are slow compared to rates of change in the capillary compartment. Third, the dissociation constant, KD, of drug binding to albumin, KA^*in vitro*^, or to AGP, KG^*in vitro*^ (Table II), as measured *in vitro*, is identical to the KA or KG in the systemic arterial compartment. This is supported by studies showing the free fraction of drug in plasma measured *in vitro* is equal to the free fraction in the systemic circulation measured *in vivo* with a microdialysis fiber implanted in the iliolumbar or jugular vein [26–30]. Fourth, drug in the red blood cell compartment is freely exchangeable with drug in the plasma compartment, as shown previously for propranolol [31] or imipramine [9]. Fifth, it is assumed that plasma proteins do not cross the BBB *in vivo* [32], in the absence of a receptor-mediated mechanism. Sixth, it is assumed there is rapid equilibrium of drug between the interstitial and intracellular compartments in brain, owing to the much greater surface area of the brain cell membrane as compared to the BBB. The surface area of the BBB is 120 cm^2^/g [33], whereas the surface area of cells in brain has been estimated at values ranging from 1,200 cm^2^/g [34] to 19,000 cm^2^/g [35]. Seventh, it is assumed that drug sequestration within the brain capillary endothelium is minor compared to drug sequestration within the post-vascular brain, because the volume of the intra-endothelial compartment, 0.8 uL/gram [36], is nearly 1,000-fold smaller than the VT of brain.

### Basal Propranolol and Imipramine Parameter Values

The total plasma drug concentration after IV infusion is approximately 100 nM in humans for propranolol or imipramine (Table II), which is considered a pharmacologic concentration in plasma [3]. The total concentration of the 67 kDa albumin in human plasma is 5.4 g/100 mL, which approximates 800 uM, and the total concentration of the 42 kDa AGP in human plasma is 0.8 mg/mL, which approximates 20 uM [12]. In subjects with metastatic cancer, the plasma albumin and AGP concentrations are 600 uM, and 70 uM, respectively [12]. The dissociation constant of albumin binding of propranolol and imipramine *in vitro*, KA^*in vitro*^, is 290 uM [8] and 42 uM [13], respectively. The dissociation constant of AGP binding of propranolol and imipramine *in vitro*, KG^*in vitro*^, is 3.3 uM [8] and 1.2 uM [14], respectively. The total concentration, PT, of the drug binding tissue protein in brain is estimated from the kinetics of propranolol sequestration in brain *in vivo* [17] and by simulations (Results). Values for the volume of the brain capillary compartment, VP, and the volume of the brain extravascular space, VT, have been described previously [15]. The rate constant (k1, min^−1^) of drug dissociation from AGP *in vivo* in the brain capillary compartment is estimated from the product of k2·KG^*in vivo*^, where k2 is the rate constant (nM^−1^ min^−1^) of drug association with AGP *in vivo* in the brain capillary compartment, and KG^*in vivo*^ is the dissociation constant (nM) of drug binding to AGP *in vivo* within the brain capillary volume. The KG^*in vivo*^ in the brain capillary compartment, or k1/k2 ratio, for propranolol and imipramine is 19 uM and 90 uM, respectively [8, 9]. The k2 value for either drug is set at 0.06 nM^−1^ min^−1^, which is equivalent to 10^6^ M^−1^ s^−1^, as this value was reported for human AGP binding of bupivacaine [16]. Bupivacaine, propranolol, and imipramine are lipophilic amine drugs. Similar to propranolol and imipramine, bupivacaine undergoes PMU via the AGP-bound pool *in vivo* in the brain capillary compartment [37]. Simulation studies (Results) show the association and dissociation rate constants *in vivo* within the brain capillary can vary 100-fold without changing the results. The rate constant (k7, min^−1^) of drug dissociation from albumin *in vivo* in the brain capillary compartment is estimated from the product of k8·KA^*in vivo*^, where k8 is the rate constant (nM^−1^ min^−1^) of drug association with albumin *in vivo* in the brain capillary compartment, and KA^*in vivo*^, is the dissociation constant of drug binding to albumin *in vivo* within the brain capillary. The KA^*in vivo*^ in the brain capillary compartment *in vivo*, which is identical to the k7/k8 ratio, for propranolol and imipramine is 290 uM and > 1,000 uM, respectively [8, 9]. The k8 value for either drug is set at 0.006 nM^−1^ min^−1^, which is equivalent to 10^5^ M^−1^ s^−1^, as this value for the association rate constant has been reported for albumin binding of multiple drugs [18], including imipramine [19]. The dissociation constant, KP (nM), of drug binding to brain tissue proteins, is computed from the k6/k5 ratio, where k6 is the rate constant of drug dissociation from brain tissue binding proteins (min^−1^), and k5 is the rate constant of drug association with brain tissue binding proteins (nM^−1^ min^−1^). The value for k6 for propranolol *in vivo* is 0.52 min^−1^ [17]. The k6 value for imipramine is ≤ 0.5 min^−1^, as imipramine sequestration in brain is greater than that of propranolol [9, 17]. The value for k5 is estimated from simulation studies (Results) guided by the experimental observation that the k5·PF product in brain *in vivo* for propranolol is 1.8 min^−1^ [17], where PF is the concentration of unbound brain tissue binding protein (Table I). The rate constant of drug metabolism in brain, k9, is set to zero as prior work shows minimal metabolism of either propranolol or imipramine in brain [17, 20]. Simulation studies examine the effect of drug metabolism in brain as reflected in changes in the k9 parameter (Results). The rate constant of brain capillary plasma flow, k10 = 60 min^−1^, is derived from a brain capillary transit time of 1 s [15]. Simulation studies vary k10 to examine the effects of changing cerebral blood flow on brain drug delivery (Results). The rate constant, k3 (min^−1^), defines drug influx from plasma to brain across the BBB, and this process is saturable both *in vivo* [17], and in isolated brain microvessels [38], for lipophilic amines such as propranolol or imipramine. The k3 value is derived from the PS/F ratio, where PS is the BBB permeability-surface area product (uL/min/gram), and F is the rate of cerebral blood flow (uL/min/gram). The PS/F ratio, which is equal to k3/k10 [8], is 1.1 and 2.5, respectively, for propranolol and imipramine [8, 9], and corresponds to k3 values of 66 min^−1^ and 150 min^−1^, given k10 = 60 min^−1^ (Table II). The rate constant, k4 (min^−1^), of drug efflux from brain to plasma across the BBB is defined as k4 = k3·(VP/VT), which assumes symmetric transport of drug in the plasma-to-brain and brain-to-plasma directions. Active efflux of drug from brain-to-plasma would produce a higher k4 value, and this is examined in simulations (Results). The parameters of plasma PK of propranolol in Table II are derived from human studies following the oral administration of 80 mg propranolol in 70 kg subjects [21, 22], which is equal to a dose of 4,600 nmol/kg (Table II).

## Results

The steady state simulations for propranolol delivery to brain are given in Table III for 9 of the 11 variables shown in Table I. The concentration of unbound AGP in the systemic compartment (GF0) or capillary compartment (GF) are not shown, as these approximate the total AGP (GT0), since GT0 >  > LT0. The basal propranolol parameters in Table II were used to produce the results for simulation 1 (Table III), where the KG^*in vivo*^ of propranolol binding to AGP is 19 uM, as compared to the KG^*in vitro*^ of 3.3 uM (Methods). The KG^*in vivo*^ of 19 uM produces a 102% increase in the capillary free (bioavailable) drug, LF, relative to the free drug in plasma *in vitro*, LF0. The free drug in brain, LM, is equal to the free drug in the capillary plasma, LF, and is also 102% greater than LF0. Owing to the enhanced dissociation of propranolol from AGP *in vivo* in the brain capillary, the capillary GL concentration decreases 61% relative to the systemic GL0 (Table I, simulation 1). As there is no enhanced dissociation of propranolol from albumin, the capillary AL concentration increases 98% relative to the systemic AL0, as the albumin captures drug dissociated from AGP (Table I, simulation 1). In simulation 2, there is no enhanced dissociation of propranolol from AGP, where KG^*in vivo*^ = KG^*in vitro*^ = 3.3 uM. In simulation 2, k1 = 198 min^−1^, which was computed from k1 = k2·KG^*in vitro*^ given a k2 value of 0.06 nM^−1^ min^−1^ (Table II). In simulation 2, the concentration of free drug *in vitro*, LF0, the concentration of free drug in the brain capillary, LF, and the concentration of free drug in brain, LM, are identical. In simulation 3, the basal value for KG^*in vivo*^, 19 uM, is used (Table II), and the plasma albumin and AGP concentrations are set at the levels observed in metastatic cancer, AF = 600 uM and GT0 = 70 nM [12]. In this setting, the concentration of free drug in the brain capillary *in vivo*, LF, and the free drug in brain, LM, are increased 258% relative to the free drug *in vitro*, LF0 (Table III, simulation 3). In simulation 4, the KG^*in vivo*^ is set at 19 uM, but the k1 and k2 values are both increased tenfold relative to simulation 1; the k2 value of 0.6 nM^−1^ min^−1^ corresponds to an association rate constant of 10^7^ M^−1^ s^−1^. In simulation 5, the KG^*in vivo*^ is set at 19 uM, but the k1 and k2 values are both decreased tenfold relative to simulation 1; the k2 value of 0.006 nM^−1^ min^−1^ corresponds to an association rate constant of 10^5^ M^−1^ s^−1^. Despite the 100-fold variation in k1 and k2 values, simulations 4 and 5 show increases in LF and LM, relative to LF0, which are comparable to simulation 1. Simulation 6 represents the dissociation-limited model of transport, which posits very high membrane permeability of the drug, relative to rates of drug dissociation from the plasma protein *in vivo*. The dissociation rate constant for propranolol binding to AGP within the brain capillary in simulation 6, k1 = 198 min^−1^, is the same as in simulation 2. Simulation 6 increases membrane permeability, k3 and k4, 1,000-fold. The k3 value of 66,600 min^−1^ corresponds to a propranolol PS/F ratio of 1,100. However, the results of simulation 6 show that a dissociation-limited model of drug transport does not allow for transport of drug into brain from the AGP or albumin bound pools in plasma, as the LF and LM values are identical to LF0 in either simulation 6 or simulation 2 (Table III). Simulation 7 represents the case of both enhanced dissociation of drug from AGP in the brain capillary compartment, as in the case of simulation 1, but in the presence of an active efflux transport (AET) mechanism that causes a selective tenfold increase in k4, but not k3. In this setting, the concentration of LF is 102% of LF0, but the concentration of LM is reduced 90% relative to LF (simulation 7, Table III). In simulation 8, the only change from the basal state of simulation 1 is an increase in k9 from 0 to 6 min^−1^, which represents drug metabolism in brain. Metabolism causes a 17% and an 89% decrease in LF and LM concentrations, respectively (simulation 8, Table III), relative to the LF and LM concentrations in simulation 1. In simulation 9, drug is metabolized in brain in parallel with the presence of ischemia, as represented by a tenfold decrease in k10, and these combined conditions cause a 59% decrease in both the LF and LM concentrations, relative to the LF and LM concentrations in simulation 8, where drug is metabolized in the presence of normal cerebral blood flow. Simulation 10 is the case where drug is metabolized in brain, where k9 is increased from 0 to 6 min^−1^, and the drug is a substrate for an AET system, where k4 is selectively increased tenfold relative to the basal state. The combination of drug metabolism, and AET, produces a decrease in the concentrations of LF and LM of 9% and 94%, respectively, relative to the LF and LM concentrations in simulation 1, where there is no drug metabolism and no AET. In the special case where the drug is not bound by plasma protein, then LF0 = LF = LM = LT0 as shown by simulation 30 of Table S1 of the Supplementary Material.

**Table III:** Propranolol Steady State Model Simulations

No	Parameter change^a^	Arterial (nM)			Capillary (nM)			Brain (nM)			K_p,brain_
		GL0	AL0	LF0	GL	AL	LF	LM	PL	PF	
1	basal	61.7	28.2	10.2	23.6	55.8	20.6	20.6	959	4,040	9.8
2	k1 = 198 min^−1^	61.7	28.2	10.2	61.7	28.2	10.2	10.2	526	4,473	5.4
3	AF = 600 uM; GT0 = 70 nM	87.4	8.5	4.1	55.7	29.6	14.7	14.7	724	4,276	7.4
4	k1 = 11,400 min^−1^; k2 = 0.6 nM^−1^ min^−1^	61.7	28.2	10.2	22.2	56.8	21.0	21.0	973	4,026	9.9
5	k1 = 114 min^−1^; k2 = 0.006 nM^−1^ min^−1^	61.7	28.2	10.2	33.6	48.6	17.9	17.9	854	4,145	8.7
6	k1 = 198 min^−1^; k3 = 66,600 min^−1^; k4 = 940 min^−1^	61.7	28.2	10.2	61.7	28.2	10.2	10.2	526	4,473	5.4
7	k4 = 9.4 min^−1^	61.7	28.2	10.2	23.6	55.8	20.6	2.1	116	4,883	1.2
8	k9 = 6 min^−1^	61.7	28.2	10.2	20.2	46.5	17.1	2.3	130	4,869	1.3
9	k9 = 6 min^−1^; k10 = 6 min^−1^;	61.7	28.2	10.2	7.61	19.23	6.96	0.95	54	4,946	0.55
10	k4 = 9.4 min^−1^; k9 = 6 min^−1^	61.7	28.2	10.2	21.9	51.2	18.8	1.2	66	4,934	0.67

^a^Basal parameters are given in Table I and are used in simulation 1; for simulations 2–10, all parameters are the basal parameters except for the parameters listed. Parameters are defined in Table I, and shown in Fig. 1

**Fig. 2:**
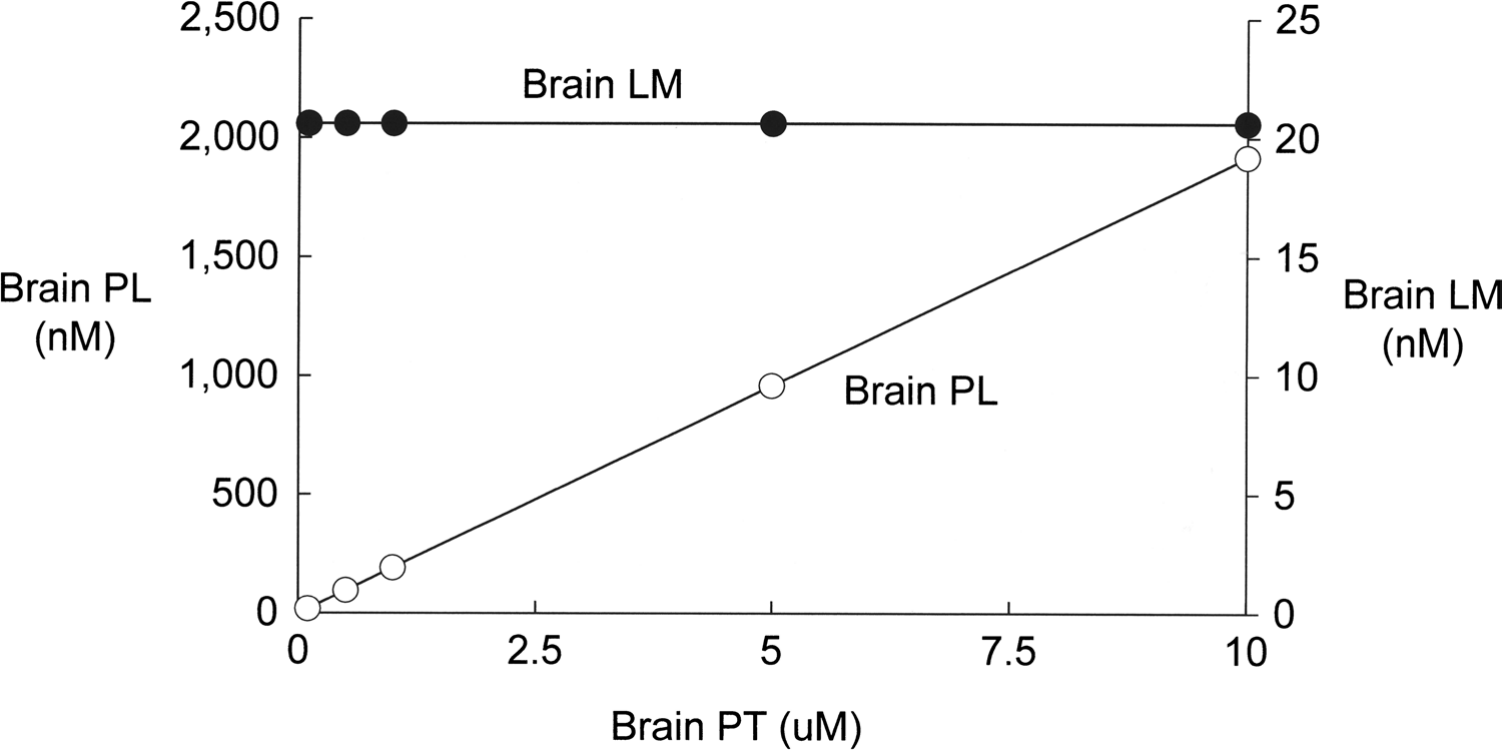
The steady state model for propranolol computes the values for free drug in brain, LM, and protein-bound drug in brain, PL, relative to the total brain concentration of drug binding tissue protein, PT, over a PT range of 0.1 to 10 uM.

The dissociation rate constant of propranolol binding to brain protein, k6 (Fig. 1), has been measured *in vivo*, and is 0.52 min^−1^ [17]. The k5·PF product of propranolol binding to brain proteins *in vivo* is 1.8 min^−1^ [17]. A k5 value of 0.006 nM^−1^ min^−1^ (Table II), in parallel with a PT value of 5,000 nM (Table II), produces a K_p,brain_ of 9.8 for propranolol (simulation 1, Table III), which matches the experimentally observed K_p,brain_ for propranolol in brain, 9.7, following an IV injection of 7.5 mg/kg [39]. Simulations listed in Table S1 of the Supplementary Material show that reduction of PT to either 1,000 nM, or 500 nM, produces progressively reduced K_p,brain_ values of 2.1 and 1.2, respectively, which are low compared to observed K_p,brain_ value for propranolol [39]. Other simulations listed in Table S1 of the Supplementary Material show the use of a k5 value of either 0.06 or 0.0006 nM^−1^ min^−1^ produced K_p,brain_ values that were too high, or too low, respectively, compared to the observed K_p,brain_ for propranolol [39]. The effect of varying the PT value was evaluated by simulations that used the basal values in Table II, but varied the PT value from 100 nM to 10,000 nM, and the predicted values for LM and PL are given in Fig. 2. Increasing PT in brain resulted in a progressive increase in the drug pool bound to brain proteins, PL, but had no effect on the concentration of free drug in brain, LM (Fig. 2). This result is anticipated by Eq. 5 (Methods), which shows the LM variable is independent of drug tissue binding parameters, PT or KP (k6/k5).

Imipramine undergoes enhanced dissociation from both albumin and AGP binding sites within the brain capillary [9], as reflected by the KG^*in vivo*^ of 90 uM [9] and the KA^*in vivo*^ > 1,000 uM [9], as compared to the KG^*in vitro*^ and KA^*in vivo*^ values of 1.2 uM [14] and 42 uM [13], respectively. The results for simulations of imipramine distribution in brain using the steady state model equations and the imipramine parameters are given in Table IV. Using the basal parameters for imipramine listed in Table II, the free (bioavailable) imipramine in brain capillary plasma, LF, and the free imipramine in brain, LM, are 18-fold greater than the free drug *in vitro*, LF0 (simulation 11, Table IV). Simulation 12 represents the case where there is no enhanced dissociation of imipramine from either albumin or AGP, and the KG^*in vivo*^ and the KA^*in vivo*^ values are fixed at the KG^*in vitro*^ and KA^*in vitro*^ values. For simulation 12, k1 is reduced to 72 min^−1^, and k1 was computed from k1 = k2·KG^*in vitro*^, where k2 = 0.06 nM^−1^ min^−1^ and KG^*in vitro*^ = 1.2 uM (Table II). In simulation 12, k7 was reduced to 252 min^−1^, where k7 was computed from k7 = k8·KA^*in vitro*^, where k8 = 0.006 nM^−1^ min^−1^ and KA^*in vitro*^ = 42 uM (Table II). Eliminating enhanced dissociation of imipramine from albumin and AGP lowers the LF and LM concentrations in brain *in vivo* to the free drug concentration *in vitro*, LF0 (simulation 12, Table IV). The K_p,brain_ value for simulation 11, 19.3, approximates the experimentally observed K_p,brain_, 23, for imipramine *in vivo* in the mouse [40], which is 12-fold higher than the K_p,brain_ predicted for simulation 12 (Table IV). The K_p,brain_ for imipramine for the rat ranges from 26–30 [41, 42]. In simulation 13, the AF and GT0 concentrations observed in metastatic cancer are used (Table IV). These changes in plasma protein concentrations produce a 31-fold increase in LF and LM *in vivo*, relative to the free drug *in vitro*, LF0 (simulation 13, Table IV). In simulations 14 and 15, the rate constants of imipramine dissociation, k1, and association, k2, with AGP are either increased or decreased tenfold, respectively, relative to the basal values in Table II, and this has only a minimal effect on the LF and LM concentrations relative to the values in simulation 11 (Table IV). Simulation 16 represents the cases of a dissociation-limited transport mechanism, where the rate constants of imipramine dissociation from AGP, k1, and from albumin, k7, are much lower than the rate constant of drug transport through the BBB, given by k3 and k4. In simulation 16, the k1 and k7 values are the same as in simulation 12, but the k3 and k4 values are increased 1,000-fold to k3 = 150,000 min^−1^ and k4 = 2,100 min^−1^. However, these very high membrane permeation rates for imipramine used for simulation 16 produced variable concentrations identical to simulation 12, and the *in vivo* concentrations of LF and LM were equal to the *in vitro* concentration of free drug, LF0 (Table IV). In simulation 17, the k4 of imipramine efflux is selectively increased tenfold to simulate transport via an AET system at the BBB, as there is evidence that imipramine is a substrate for p-glycoprotein [43]. This simulation caused no change in the drug concentration in the brain capillary compartment, LF, but resulted in a 90% reduction in the LM concentration, relative to simulation 11 (Table IV). In simulation 18, drug metabolism was modeled as the value for k9 was increased from 0 to 6 min^−1^. This produced a 48% and 86% decrease in the LF and LM concentrations as compared to simulation 11 (Table IV). In simulation 19, drug metabolism was combined with ischemia, as represented by a tenfold decrease in k10, and this resulted in a 90% and 97% decrease in the LF and LM concentrations as compared to simulation 11. In simulation 20, AET and drug metabolism were combined with a tenfold increase in k4 and an increase in k9 from 0 to 6 min^−1^. This produced a 22% and 94% decrease in the LF and LM concentrations, respectively, compared to simulation 11 (Table IV). A summary of all simulations for brain delivery of imipramine is given in Table S2 of the Supplementary Material.

**Table IV:** Imipramine Steady State Model Simulations

No	Parameter change^a^	Arterial (nM)			Capillary (nM)			Brain (nM)			K_p,brain_
		GL0	AL0	LF0	GL	AL	LF	LM	PL	PF	
11	basal	45.3	51.9	2.73	11.3	39.5	49.2	50.2	1,880	3,120	19.3
12	k1 = 72 min^−1^ k7 = 252 min^−1^	45.3	51.9	2.73	45.3	51.9	2.73	2.78	162	4,838	1.6
13	AF = 600 uM; GT0 = 70 nM	79.2	19.4	1.36	33.1	25.1	41.9	42.7	1,695	3,305	17.4
14	k1 = 54,000 min^−1^; k2 = 0.6 nM^−1^ min^−1^	45.3	51.9	2.73	11.0	39.6	49.4	50.3	1,884	3,116	19.3
15	k1 = 540 min^−1^; k2 = 0.006 nM^−1^ min^−1^	45.3	51.9	2.73	14.1	38.3	47.6	48.6	1,843	3,157	18.9
16	k1 = 72 min^−1^; k3 = 150,000 min^−1^; k4 = 2,100 min^−1^ k7 = 252 min^−1^;	45.3	51.9	2.73	45.3	51.9	2.73	2.77	161	4,839	1.6
17	k4 = 21 min^−1^	45.3	51.9	2.73	11.3	39.5	49.2	5.02	284	4,716	2.9
18	k9 = 6 min^−1^	45.3	51.9	2.73	6.13	20.8	25.6	6.78	376	4,624	3.8
19	k9 = 6 min^−1^; k10 = 6 min^−1^	45.3	51.9	2.73	1.13	3.94	4.86	1.29	76	4,924	0.77
20	k4 = 21 min^−1^; k9 = 6 min^−1^	45.3	51.9	2.73	8.97	31.1	38.6	3.06	177	4,823	1.8

^a^Basal parameters are given in Table II and are used in simulation 11; for simulations 12–20, all parameters are the basal parameters except for the parameters listed

**Fig. 3:**
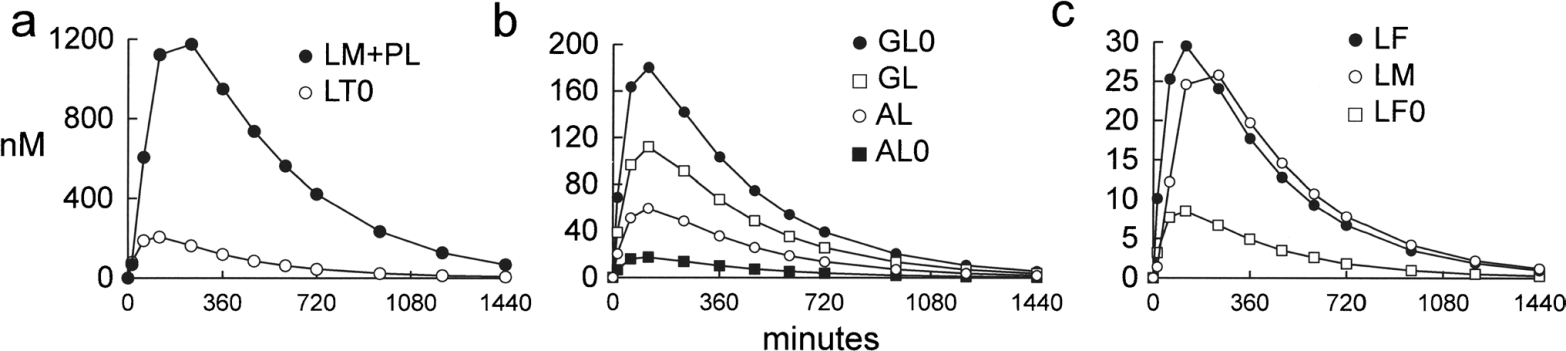
The non-steady state model for propranolol computes the concentrations for the total drug in brain (LM + PL) and total drug in plasma (LT0) (panel **a**), for the concentrations of albumin-bound drug and AGP-bound drug in the systemic compartment (AL0, GL0) and brain capillary compartment (AL, GL) (panel **b**), and the concentrations of free (bioavailable) drug in the brain capillary (LF), the concentration of free drug in brain (LM), and the concentration of free drug *in vitro* (LF0) (panel **c**).

The non-steady state model was used to simulate variable concentrations over 1440 min after a single oral ingestion of 80 mg propranolol in humans. The total plasma concentration of drug, LT0[t], was computed from Eq. 13 using the PK parameters in Table II, and the plasma LT0 concentrations are plotted in Fig. 3a. The simulations were performed with the basal propranolol parameters in Table II, except the AF = 600 uM and the GT0 = 70 nM as in simulation 3 (Table III). The values for globulin bound drug and albumin bound drug in the arterial compartment, GL0 and AL0, and brain capillary compartment, GL and AL, are plotted in Fig. 3b. The concentration of free (bioavailable) drug in the brain capillary, LF, the free drug in brain, LM, and the free drug *in vitro*, LF0, are plotted in Fig. 3c. The sum of the LM and PL concentrations are plotted in Fig. 3a. The (LM + PL)/LT0, or K_p,brain_ values, are 5.4, 7.2, 8.0, and 9.1, at 2, 4, 6, and 10 h after administration and reached equilibrium K_p,brain_ values of 10 between 12–24 h after administration. The plasma area under the concentration curve (AUC) for each variable was determined with the trapezoid method, and these values are given in Table V for the arterial, brain capillary, and brain compartments. The AUC for the brain capillary unbound (bioavailable) drug, LF, and unbound drug in brain, LM, are 260% greater than the AUC for unbound drug *in vitro*, LF0 (Table V). These results on the comparative values of the AUC for LF, LM, and LF0 with the non-steady state model are identical to the comparative values for concentrations for LF, LM, and LF0 with the steady state model (simulation 3, Table III). The ratio of AUC for the LM + PL pools, relative to the LT0 pool, is 7.4. The total propranolol plasma concentration, LT0, used in the steady state model, 100 nM (Table II), is reached at 360–480 min after PO administration (Fig. 3a). At this time, the concentrations of LF0, LF, and LM (Fig. 3c), approximate the same concentrations generated with the steady state model for these variables (simulation 3, Table III). The AUC values for the globulin-bound and albumin-bound drug in the arterial and brain capillary compartments are inverted, owing to the selective enhanced dissociation of propranolol from AGP, but not from albumin, within the capillary compartment relative to the arterial compartment of brain.

**Table V:** Propranolol AUC in Arterial, Brain Capillary, and Brain Compartments

Compartment	Variable (nM)	AUC (nmol·min/L)
Arterial	LT0	48,489
	LF0	1,994
	GL0	42,373
	AL0	4,136
Brain capillary	LF	7,122
	GL	27,059
	AL	14,379
Brain	LM	7,211
	LM + PL	359,655
	PL	352,655

AUC computed from T = 0 to T = 1,440 min with the trapezoid method based on the concentrations shown in Fig. 3. Variables are defined in Table I

## Discussion

The PBPK model described in this work focuses on the *in vivo* kinetics of drug binding to plasma proteins, albumin and AGP, and brain tissue proteins, as outlined in Fig. 1. Model parameters include the individual association rate constants (kon) and dissociation rate constants (koff) of drug binding *in vivo* to albumin and AGP in plasma, and to drug binding proteins in brain (Table II). The model does not address drug binding to the target receptor, which has been examined in prior work on the relative effects of the kon and koff rates governing the drug-receptor binding reaction [44]. The present model is based on the premise that the dissociation constant, KD^*in vitro*^, governing drug binding to the plasma protein *in vitro* may, or may not, be equal to the KD^*in vivo*^ that governs drug binding to the plasma protein at the interface of plasma and the endothelial glycocalyx *in vivo* within the brain capillary compartment. It is possible to determine the KD^*in vivo*^ within the brain capillary *in vivo* using carotid arterial injection methods [34]. Such vivo studies of drug binding to plasma proteins within the brain capillary compartment show that the KD^*in vivo*^ is generally, but not always, greater than the KD^*in vitro*^, which is indicative of enhanced dissociation of drug from the plasma protein *in vivo*, such that the protein-bound drug is available for uptake by brain, without exodus of the plasma protein, per se, from the capillary compartment. Plasma protein mediated drug uptake, or PMU [7], in brain is examined in the present PBPK model. This study models 2 drugs, propranolol and imipramine. Propranolol undergoes a modest transport into brain *in vivo* from the AGP-bound pool, but not from the albumin-bound pool [8]. In contrast, imipramine undergoes marked transport into brain *in vivo* from both the AGP-bound and albumin-bound pools [9].

The different models that have been proposed for PMU have been recently reviewed [45], and include a receptor model, a dissociation-limited model, and an enhanced dissociation model. Although both albumin and AGP bind multiple proteins [46, 47], no gene encoding a specific albumin or AGP receptor has been cloned to date. Both *in vivo* investigations [48], and *in vitro* studies with isolated brain microvessels [49], show no specific albumin receptor at the BBB. In the dissociation-limited model, it is posited that membrane permeability is actually 2–3 log orders higher than experimentally observed values, and in this setting, drug is rapidly transported into the organ following dissociation from the plasma protein within the capillary compartment, rather than undergo re-association with the plasma protein [50]. While there is no experimental basis for the postulate of extremely high PS products for drug transport at the BBB, the dissociation-limited model is examined in simulations 6 and 16 for propranolol and imipramine, respectively (Tables III-IV). These simulations show that there is no transport from the plasma protein-bound pool even if BBB permeability to the drug (k3, k4) was increased 1,000-fold over experimentally observed values.

The observation, from several separate laboratories, is that the KD^*in vivo*^, within the brain capillary compartment, is often times much greater than the KD^*in vitro*^ [34]. The KD is a measure of ligand affinity for the protein, and this affinity is generally dictated by rates of ligand dissociation, which are a function of the conformation of the protein binding site [51, 52]. Both albumin and AGP undergo conformational changes upon contact with biomembrane surfaces [53–56]. Within the brain capillary, the major surface exposed to circulating plasma proteins is the endothelial glycocalyx (EG). The EG is rich in proteoglycans and glycosaminoglycans [57], which bind albumin and AGP [58–60]. The EG is 400 nm thick at the brain microcirculation [61, 62]. Therefore, the thickness of the EG at the brain capillary endothelium is greater than the thickness of the brain endothelial cell, which is about 300 nm [63]. The EG covers about 40% of the surface of the endothelium in the brain, as compared to only 4% of the endothelial surface in the lung [62]. Potential interaction between the EG and plasma proteins is rarely discussed in investigations of brain delivery of drugs bound to plasma proteins.

The investigations demonstrating an increase in the KD^*in vivo*^, relative to the KD^*in vitro*^, of drug binding to albumin or AGP have been conducted with human albumin and human AGP [34]. There may be important differences in drug binding to plasma proteins from non-human species, particularly rats and mice. The amino acid sequence of rat serum albumin (RSA, AAH85359) or mouse serum albumin (MSA, AAH49971) is 72–73% identical to the sequence of human serum albumin (HSA, AAA98797). Owing to these sequence differences, the diazepam binding site on HSA is not present on RSA [64], and the affinity of RSA for diazepam is 30-fold lower as compared to diazepam binding to HSA [65]. The amino acid sequence of rat AGP (NP_445740) or mouse AGP (NP_032794) is only 49–50% identical to the sequence of human AGP (AAA35515). In addition, the plasma concentration of AGP in the rat [66] or mouse [67] is more than tenfold lower than the concentration of AGP in human plasma, which is about 1 mg/mL [12, 68]. There is little information on the extent to which drugs such as propranolol or imipramine, or other CNS drugs, are bound to rat or mouse albumin or to rat or mouse AGP, as compared to the human proteins [69].

The principal lines of evidence against plasma protein mediated uptake are experiments with cerebral microdialysis, which are generally performed in rats or mice. The unbound drug in the brain microdialysate corresponds to the unbound drug in plasma measured *in vitro* in the case of brain microdialysis of diazepam [26], imipramine [43], or propranolol [70]. These studies in the rat are assessments of drug binding to rat albumin or rat AGP. Apart from the role of species effects in plasma protein binding of drugs, studies with microdialysis are confounded by the brain penetration injury and brain ischemia induced by the implantation in brain of a dialysis fiber [71–73]. Brain ischemia is induced in the region around the fiber, because the diameter of the dialysis fiber, 200–600 microns, is much greater than the inter-capillary distance in brain, which is about 40 microns [74]. The IV administration of fluorescent 100 nm microspheres shows no perfusion of capillaries in the region contiguous with the dialysis fiber implanted in brain [71]. The fluid within the dialysis fiber implanted in brain may be in equilibrium with the extracellular space (ECS) up to 2,000 microns removed from the dialysis fiber for a poorly diffusible compound confined largely to the ECS [75]. However, for molecules such as lipophilic amine drugs, which are highly diffusible across cell membranes and are sequestered within brain cells, the dialysis fiber may be in equilibrium with brain ECS up to only 100–200 microns removed from the dialysis fiber [75], a region within the ischemic zone surrounding the fiber. Both brain injury and/or brain ischemia cause rapid shedding of the EG at the brain capillary within 60 min of the insult, and this shedding of the EG in brain persists for at least 7 days [76–78]. If the implantation in brain of a dialysis fiber causes shedding of the EG and if PMU is initiated by plasma protein interaction with the EG, then no PMU may be detectable with cerebral microdialysis.

The PBPK model used in this investigation also examines drug binding/sequestration within the brain compartment (Fig. 1). Drug sequestration in brain produces a K_p,brain_ value > 1, which is the case both for propranolol, where the K_p,brain_ is 10 [39], and imipramine, where the K_p,brain_ is 23 [40]. The brain sequestration of lipophilic amines, such as propranolol or imipramine, which have high pKa values, and which are protonated at physiologic pH, may be due binding to anionic phospholipids, sequestration within the acidic compartment of the lysosome, which constitutes 1–3% of the cell volume [79], or binding to brain microsomal or mitochondrial proteins. The unbound volume of distribution, V_u_, of propranolol and atenolol in brain slices is 112 mL/g and 2.5 mL/g, respectively [80]. Since both propranolol and atenolol have a pKa of 9.5–9.6 [81], the binding of either drug to acidic phospholipids, or lysosomal entrapment, should be comparable. The 45-fold higher V_u_ for propranolol as compared to atenolol in brain slices [80] suggests the major mechanism for the selective sequestration in brain of propranolol, as compared to atenolol, is binding to brain tissue proteins. The absence of atenolol binding to brain proteins is confirmed by the f_u,brain_ = 1.0 in brain homogenate, and the low K_p,brain_ of 0.04 for atenolol [82]. In contrast, the f_u,brain_ for propranolol or imipramine is low 0.01–0.02 in brain homogenate [83], and both propranolol and imipramine are bound, via a saturable mechanism, by tissue microsomal proteins [84]. An early study showed the saturable binding of propranolol to brain tissue proteins was equally distributed to the microsomal and mitochondrial fractions of brain cells [39], and these subcellular compartments comprise 73% of the total cellular protein [85]. Drug sequestration in tissue homogenates *in vitro* is inversely related to the f_u,tissue_, and the sum of f_u,tissue_ in multiple organs is predictive of the systemic volume of distribution [86, 87].

Drug binding to brain tissue proteins is directly related to K_p,brain_, and this is demonstrated in Fig. 2, where the brain concentration of protein-bound drug, PL, is directly related to the total brain tissue protein concentration, PT. The data in Fig. 2 also shows that the concentration of the free drug in brain, LM, or C_u,brain_, is independent of the PT concentration. The independence of the free drug in brain on drug binding to tissue proteins is predicted by Eq. 7 (Methods), which shows, in the absence of drug metabolism, where k9 = 0, that the free drug in brain, LM, is a function only of the bioavailable drug in plasma, LF, and bi-directional BBB transport. Equation 7 shows the LM in brain is proportional to the concentration of the free (bioavailable) drug in plasma, LF. The LM/LF ratio, or C_u,brain_/C_u,plasma_, constitutes the k_p,uu,brain_ parameter (Methods), which, similar to C_u,brain_, is also independent of drug partitioning in brain, such as binding to tissue proteins. However, the k_p,uu,brain_ parameter is typically measured *in vitro* by equilibrium dialysis (ED) of aliquots of plasma, for determination of C_u,plasma_, and brain homogenate, for determination of C_u,brain_. The C_u,brain_ measured *in vitro* by ED of brain homogenate is inversely related to drug binding to brain tissue proteins, but the C_u,brain_ *in vivo* is independent of drug binding to brain tissue proteins (Fig. 2). *In vitro* measurements of C_u,plasma_ by ED will underestimate the unbound (bioavailable) drug in plasma in brain *in vivo*, if PMU takes place at the brain endothelial interface.

## Conclusion

The PBPK model described in this work examines brain delivery of plasma protein bound drugs by simulating the effects of changes in multiple kinetic parameters on drug binding to albumin and AGP *in vivo* in the brain capillary compartment, and to tissue proteins in the brain compartment (Fig. 1). The results of this study highlight the limitations of the *in vitro* measurement of the free drug in plasma or brain by equilibrium dialysis of plasma or brain homogenate, respectively. Equilibrium dialysis of plasma will under-estimate the free drug in brain if there is significant PMU of the plasma protein-bound drug. Equilibrium dialysis of brain homogenate is a function of drug binding to tissue proteins *in vitro*, whereas the free drug in brain *in vivo* is independent of drug binding to tissue proteins. The free drug in brain is a function of the free (bioavailable) drug in the brain capillary compartment, bi-directional BBB drug transport, and brain metabolism of the drug.

## Supplementary Information

Below is the link to the electronic supplementary material.

Supplementary file1 (DOCX 47 KB)
