# Supplementary material for: Physiologically Based Pharmacokinetic Model of Brain Delivery of Plasma Protein Bound Drugs
Source: Pharm Res. 2023 Feb 24;40(3):661–74. doi: 10.1007/s11095-023-03484-2 (PMC10036418; doi:10.1007/s11095-023-03484-2)
Supplement: Supplementary file 1 — Supplementary file1 (DOCX 47 KB) [file 11095_2023_3484_MOESM1_ESM.docx]

**SUPPLEMENTARY MATERIAL**

**Physiologically Based Pharmacokinetic Model of Brain Delivery of**

**Plasma Protein Bound Drugs**

William M. Pardridge^1^

^1^UCLA, Los Angeles, CA 90095

Email: [wpardrid@ucla.edu](mailto:wpardrid@ucla.edu)

ORCID: <https://orcid.org/0000-0002-2664-1338>

| **Supplementary Table S1.**  **Brain propranolol (Mathematica Solve-steady state model)**  *Yellow highlight indicates change in parameter relative to simulation 1* | | | | | | | | | | | |
| --- | --- | --- | --- | --- | --- | --- | --- | --- | --- | --- | --- |
| Parameter/Variable | units | Simulation number (simulation number in Table III) | | | | | | | | | |
|  |  | 1 (1) | 2 (2) | 3 | 4 | 5 | 6 | 7 | 8 | 9 | 10 |
| **k1** | min^-1^ | 1140 | 198 | 1140 | 1140 | 1140 | 1140 | 1140 | 1140 | 1140 | 1140 |
| **k2** | nM^-1^min^-1^ | 0.06 | 0.06 | 0.06 | 0.06 | 0.06 | 0.06 | 0.06 | 0.06 | 0.06 | 0.06 |
| **k3** | min^-1^ | 66 | 66 | 66 | 66 | 66 | 66 | 66 | 66 | 66 | 66 |
| **k4** | min^-1^ | 0.943 | 0.943 | 0.943 | 0.943 | 0.943 | 0.943 | 0.943 | 0.943 | 0.943 | 0.943 |
| **k5** | nM^-1^min^-1^ | 0.006 | 0.006 | 0.006 | 0.006 | 0.006 | 0.006 | 0.0006 | 0.0006 | 0.0006 | 0.06 |
| **k6** | min^-1^ | 0.52 | 0.52 | 0.52 | 0.52 | 0.52 | 0.52 | 0.52 | 0.52 | 0.52 | 0.52 |
| **k7** | min^-1^ | 1740 | 1740 | 1740 | 1740 | 1740 | 1740 | 1740 | 1740 | 1740 | 1740 |
| **k8** | nM^-1^min^-1^ | 0.006 | 0.006 | 0.006 | 0.006 | 0.006 | 0.006 | 0.006 | 0.006 | 0.006 | 0.006 |
| **k9** | min^-1^ | 0 | 0 | 0 | 0 | 0 | 0 | 0 | 0 | 0 | 0 |
| **k10** | min^-1^ | 60 | 60 | 60 | 60 | 60 | 60 | 60 | 60 | 60 | 60 |
| **KG (p11)** | nM | 3,300 | 3,300 | 3,300 | 3,300 | 3,300 | 3,300 | 3,300 | 3,300 | 3,300 | 3,300 |
| **KA (p12)** | nM | 290,000 | 290,000 | 290,000 | 290,000 | 290,000 | 290,000 | 290,000 | 290,000 | 290,000 | 290,000 |
| **GT0 (p13)** | nM | 20,000 | 20,000 | 20,000 | 20,000 | 20,000 | 20,000 | 20,000 | 20,000 | 20,000 | 20,000 |
| **AF (p14)** | nM | 800,000 | 800,000 | 800,000 | 800,000 | 800,000 | 800,000 | 800,000 | 800,000 | 800,000 | 800,000 |
| **PT (p15)** | nM | 5,000 | 5,000 | 10,000 | 1,000 | 500 | 100 | 5,000 | 10,000 | 1,000 | 5,000 |
| **LT0 (p16)** | nM | 100 | 100 | 100 | 100 | 100 | 100 | 100 | 100 | 100 | 100 |
| **VP (p17)** | L/kg | 0.01 | 0.01 | 0.01 | 0.01 | 0.01 | 0.01 | 0.01 | 0.01 | 0.01 | 0.01 |
| **VT (p18)** | L/kg | 0.7 | 0.7 | 0.7 | 0.7 | 0.7 | 0.7 | 0.7 | 0.7 | 0.7 | 0.7 |
| **GL0** | nM | 61.65 | 61.65 | 61.65 | 61.65 | 61.65 | 61.65 | 61.65 | 61.65 | 61.65 | 61.65 |
| **AL0** | nM | 28.15 | 28.15 | 28.15 | 28.15 | 28.15 | 28.15 | 28.15 | 28.15 | 28.15 | 28.15 |
| **LF0** | nM | 10.2 | 10.2 | 10.2 | 10.2 | 10.2 | 10.2 | 10.2 | 10.2 | 10.2 | 10.2 |
| **GL** | nM | 23.63 | 61.65 | 23.63 | 23.63 | 23.63 | 23.63 | 23.63 | 23.63 | 23.63 | 23.63 |
| **AL** | nM | 55.8 | 28.15 | 55.8 | 55.8 | 55.8 | 55.8 | 55.8 | 55.8 | 55.8 | 55.8 |
| **LF** | nM | 20.57 | 10.2 | 20.57 | 20.57 | 20.57 | 20.57 | 20.57 | 20.57 | 20.57 | 20.57 |
| **GF** | nM | 19,976 | 19,938 | 19,976 | 19,938 | 19,976 | 19,976 | 19,976 | 19,976 | 19,976 | 19,976 |
| **LM** | nM | 20.57 | 10.20 | 20.57 | 20.57 | 20.57 | 20.57 | 20.57 | 20.57 | 20.57 | 20.57 |
| **PL** | nM | 959 | 526 | 1918 | 192 | 96 | 19.2 | 116 | 231 | 23.2 | 3518 |
| **PF** | nM | 4,040 | 4,473 | 8081 | 808 | 404 | 81 | 4884 | 9768 | 977 | 1482 |
| **LM+PL** | nM | 979.57 | 536.2 | 1939 | 213 | 117 | 40 | 137 | 252 | 44 | 3539 |
| **Kp,brain=(LM+PL)/**  **LT0** | -- | 9.8 | 5.4 | 19.4 | 2.1 | 1.2 | 0.40 | 1.4 | 2.5 | 0.44 | 35.4 |
| **In vitro fu,p=LF0/LT0** | -- | 0.102 | 0.102 | 0.102 | 0.102 | 0.102 | 0.102 | 0.102 | 0.102 | 0.102 | 0.102 |
| **In vivo fu,p=LF/LT0** | -- | 0.206 | 0.102 | 0.206 | 0.206 | 0.206 | 0.206 | 0.206 | 0.206 | 0.206 | 0.206 |
| **Capillary KG=k1/k2** | uM | 19 | 3.3 | 19 | 19 | 19 | 19 | 19 | 19 | 19 | 19 |
| **Capillary KA=k7/k8** | uM | 290 | 290 | 290 | 290 | 290 | 290 | 290 | 290 | 290 | 290 |
| **Tissue KP=k6/k5** | nM | 87 | 87 | 87 | 87 | 87 | 87 | 870 | 870 | 870 | 8.7 |

| **Supplementary Table S1 (continued)**  **Brain propranolol (Mathematica Solve-steady state model)**  *Yellow highlight indicates change in parameter relative to simulation 1* | | | | | | | | | | | |
| --- | --- | --- | --- | --- | --- | --- | --- | --- | --- | --- | --- |
| Parameter/Variable | units | Simulation number (simulation number in Table III) | | | | | | | | | |
|  |  | 11 | 12 (3) | 13 (4) | 14 (5) | 15 | 16 | 17 | 18 (7) | 19 (8) | 20 (10) |
| **k1** | min^-1^ | 1140 | 1140 | 11,400 | 114 | 198 | 19.8 | 1.98 | 1140 | 1140 | 1140 |
| **k2** | nM^-1^min^-1^ | 0.06 | 0.06 | 0.6 | 0.006 | 0.06 | 0.006 | 0.0006 | 0.06 | 0.06 | 0.06 |
| **k3** | min^-1^ | 66 | 66 | 66 | 66 | 6,600 | 6600 | 6600 | 66 | 66 | 66 |
| **k4** | min^-1^ | 0.943 | 0.943 | 0.943 | 0.943 | 94 | 94 | 94 | 9.4 | 0.943 | 9.4 |
| **k5** | nM^-1^min^-1^ | 0.006 | 0.006 | 0.006 | 0.006 | 0.006 | 0.006 | 0.006 | 0.006 | 0.006 | 0.006 |
| **k6** | min^-1^ | 0.52 | 0.52 | 0.52 | 0.52 | 0.52 | 0.52 | 0.52 | 0.52 | 0.52 | 0.52 |
| **k7** | min^-1^ | 1740 | 1740 | 1740 | 1740 | 1740 | 1740 | 1740 | 1740 | 1740 | 1740 |
| **k8** | nM^-1^min^-1^ | 0.006 | 0.006 | 0.006 | 0.006 | 0.006 | 0.006 | 0.006 | 0.006 | 0.006 | 0.006 |
| **k9** | min^-1^ | 0 | 0 | 0 | 0 | 0 | 0 | 0 | 0 | 6 | 6 |
| **k10** | min^-1^ | 60 | 60 | 60 | 60 | 60 | 60 | 60 | 60 | 60 | 60 |
| **KG (p11)** | nM | 3,300 | 3,300 | 3,300 | 3,300 | 3,300 | 3,300 | 3,300 | 3,300 | 3,300 | 3,300 |
| **KA (p12)** | nM | 290,000 | 290,000 | 290,000 | 290,000 | 290,000 | 290,000 | 290,000 | 290,000 | 290,000 | 290,000 |
| **GT0 (p13)** | nM | 20,000 | 70,000 | 20,000 | 20,000 | 20,000 | 20,000 | 20,000 | 20,000 | 20,000 | 20,000 |
| **AF (p14)** | nM | 800,000 | 600,000 | 800,000 | 800,000 | 800,000 | 800,000 | 800,000 | 800,000 | 800,000 | 800,000 |
| **PT (p15)** | nM | 5,000 | 5,000 | 5,000 | 5,000 | 5,000 | 5,000 | 5,000 | 5,000 | 5,000 | 5,000 |
| **LT0 (p16)** | nM | 100 | 100 | 100 | 100 | 100 | 100 | 100 | 100 | 100 | 100 |
| **VP (p17)** | L/kg | 0.01 | 0.01 | 0.01 | 0.01 | 0.01 | 0.01 | 0.01 | 0.01 | 0.01 | 0.01 |
| **VT (p18)** | L/kg | 0.7 | 0.7 | 0.7 | 0.7 | 0.7 | 0.7 | 0.7 | 0.7 | 0.7 | 0.7 |
| **GL0** | nM | 61.65 | 87.35 | 61.65 | 61.65 | 61.65 | 61.65 | 61.65 | 61.65 | 61.65 | 61.65 |
| **AL0** | nM | 28.15 | 8.53 | 28.15 | 28.15 | 28.15 | 28.15 | 28.15 | 28.15 | 28.15 | 28.15 |
| **LF0** | nM | 10.2 | 4.12 | 10.2 | 10.2 | 10.2 | 10.2 | 10.2 | 10.2 | 10.2 | 10.2 |
| **GL** | nM | 23.63 | 55.69 | 22.24 | 33.56 | 61.65 | 61.65 | 61.65 | 23.63 | 20.15 | 21.90 |
| **AL** | nM | 55.8 | 29.63 | 56.81 | 48.58 | 28.15 | 28.15 | 28.15 | 55.8 | 46.51 | 51.18 |
| **LF** | nM | 20.57 | 14.67 | 20.95 | 17.86 | 10.20 | 10.20 | 10.20 | 20.57 | 17.08 | 18.84 |
| **GF** | nM | 19,976 | 69,944 | 19,977 | 19,966 | 19,938 | 19,938 | 19,938 | 19,976 | 19,979 | 19,978 |
| **LM** | nM | 20.57 | 14.67 | 20.95 | 17.86 | 10.23 | 10.23 | 10.23 | 2.06 | 2.32 | 1.15 |
| **PL** | nM | 959 | 724 | 973 | 854 | 528 | 528 | 528 | 116 | 130 | 66 |
| **PF** | nM | 4,040 | 4,276 | 4,026 | 4,145 | 4,471 | 4,471 | 4,471 | 4,883 | 4,869 | 4,934 |
| **LM+PL** | nM | 979.57 | 739 | 994 | 872 | 538 | 538 | 538 | 118 | 132 | 67 |
| **Kp,brain=(LM+PL)/LT0** | -- | 9.8 | 7.4 | 9.9 | 8.7 | 5.4 | 5.4 | 5.4 | 1.2 | 1.3 | 0.67 |
| **In vitro fu,p=LF0/LT0** | -- | 0.102 | 0.041 | 0.102 | 0.102 | 0.102 | 0.102 | 0.102 | 0.102 | 0.102 | 0.102 |
| **In vivo fu,p=LF/LT0** | -- | 0.206 | 0.147 | 0.209 | 0.179 | 0.102 | 0.102 | 0.102 | 0.206 | 0.171 | 0.188 |
| **Capillary KG=k1/k2** | uM | 19 | 19 | 19 | 19 | 3.3 | 3.3 | 3.3 | 19 | 19 | 19 |
| **Capillary KA=k7/k8** | uM | 290 | 290 | 290 | 290 | 290 | 290 | 290 | 290 | 290 | 290 |
| **Tissue KP=k6/k5** | nM | 87 | 87 | 87 | 87 | 87 | 87 | 87 | 87 | 87 | 87 |

| **Supplementary Table S1 (continued)**  **Brain propranolol (Mathematica Solve-steady state model)**  *Yellow highlight indicates change in parameter relative to simulation 1* | | | | | | | | | | | |
| --- | --- | --- | --- | --- | --- | --- | --- | --- | --- | --- | --- |
| Parameter/Variable | units | Simulation number (simulation number in Table III) | | | | | | | | | |
|  |  | 21 | 22 | 23 (9) | 24 | 25 | 26 | 27 | 28 (6) | 29 | 30 |
| **k1** | min^-1^ | 198 | 0.198 | 1140 | 1140 | 1140 | 1140 | 198 | 198 | 198 | 198 |
| **k2** | nM^-1^min^-1^ | 0.06 | 0.00006 | 0.06 | 0.06 | 0.06 | 0.06 | 0.06 | 0.06 | 0.06 | 0.06 |
| **k3** | min^-1^ | 66 | 66,000 | 66 | 66 | 66 | 66 | 6,600 | 66,000 | 66,000 | 66 |
| **k4** | min^-1^ | 0.943 | 940 | 0.943 | 0.943 | 0.943 | 0.943 | 94.3 | 943 | 943 | 0.943 |
| **k5** | nM^-1^min^-1^ | 0.006 | 0.006 | 0.006 | 0.06 | 0.06 | 0.0006 | 0.006 | 0.006 | 0.006 | 0.006 |
| **k6** | min^-1^ | 0.52 | 0.52 | 0.52 | 0.52 | 0.52 | 0.52 | 0.52 | 0.52 | 0.52 | 0.52 |
| **k7** | min^-1^ | 1740 | 1740 | 1740 | 1740 | 1740 | 1740 | 1740 | 1740 | 1740 | 1740 |
| **k8** | nM^-1^min^-1^ | 0.006 | 0.006 | 0.006 | 0.006 | 0.006 | 0.006 | 0.006 | 0.006 | 0.006 | 0.006 |
| **k9** | min^-1^ | 0 | 0 | 6 | 0 | 0 | 0 | 0 | 0 | 0 | 0 |
| **k10** | min^-1^ | 60 | 60 | 6 | 60 | 60 | 60 | 60 | 60 | 60,000 | 60 |
| **KG (p11)** | nM | 3,300 | 3,300 | 3,300 | 3,300 | 3,300 | 3,300 | 3,300 | 3,300 | 3,300 | 3,300 |
| **KA (p12)** | nM | 290,000 | 290,000 | 290,000 | 290,000 | 290,000 | 290,000 | 290,000 | 290,000 | 290,000 | 290,000 |
| **GT0 (p13)** | nM | 20,000 | 20,000 | 20,000 | 20,000 | 20,000 | 20,000 | 20,000 | 20,000 | 20,000 | 20,000 |
| **AF (p14)** | nM | 800,000 | 800,000 | 800,000 | 800,000 | 800,000 | 800,000 | 800,000 | 800,000 | 800,000 | 0 |
| **PT (p15)** | nM | 5,000 | 5,000 | 5,000 | 1,000 | 500 | 5,000 | 5,000 | 5,000 | 5,000 | 0 |
| **LT0 (p16)** | nM | 100 | 100 | 100 | 100 | 100 | 100 | 100 | 100 | 100 | 100 |
| **VP (p17)** | L/kg | 0.01 | 0.01 | 0.01 | 0.01 | 0.01 | 0.01 | 0.01 | 0.01 | 0.01 | 0.01 |
| **VT (p18)** | L/kg | 0.7 | 0.7 | 0.7 | 0.7 | 0.7 | 0.7 | 0.7 | 0.7 | 0.7 | 0.7 |
| **GL0** | nM | 61.65 | 61.65 | 61.65 | 61.65 | 61.65 | 61.65 | 61.65 | 61.65 | 61.65 | 0 |
| **AL0** | nM | 28.15 | 28.15 | 28.15 | 28.15 | 28.15 | 28.15 | 28.15 | 28.15 | 28.15 | 0 |
| **LF0** | nM | 10.2 | 10.2 | 10.2 | 10.2 | 10.2 | 10.2 | 10.2 | 10.2 | 10.2 | 100 |
| **GL** | nM | 61.65 | 61.65 | 7.61 | 23.63 | 23.63 | 23.63 | 61.65 | 61.65 | 61.65 | 0 |
| **AL** | nM | 28.15 | 28.15 | 19.23 | 55.8 | 55.8 | 55.8 | 28.15 | 28.15 | 28.15 | 0 |
| **LF** | nM | 10.2 | 10.2 | 6.96 | 20.6 | 20.6 | 20.6 | 10.2 | 10.2 | 10.2 | 100 |
| **GF** | nM | 19,938 | 19,938 | 19,992 | 19,976 | 19,976 | 19,976 | 19,938 | 19,938 | 19,938 | 0 |
| **LM** | nM | 10.20 | 10.20 | 0.95 | 20.6 | 20.6 | 20.6 | 10.20 | 10.20 | 10.20 | 100 |
| **PL** | nM | 526 | 528 | 54 | 704 | 352 | 116 | 526 | 526 | 526 | 2,678 |
| **PF** | nM | 4,473 | 4,472 | 4,946 | 296 | 148 | 4,884 | 4,473 | 4,473 | 4,473 | 2,322 |
| **LM+PL** | nM | 536.2 | 538 | 55 | 725 | 375 | 137 | 536.2 | 536.2 | 536.2 | 2,778 |
| **Kp,brain=(LM+PL)/LT0** | -- | 5.4 | 5.4 | 0.55 | 7.2 | 3.8 | 1.4 | 5.4 | 5.4 | 5.4 | 27.8 |
| **In vitro fu,p=LF0/LT0** | -- | 0.102 | 0.102 | 0.102 | 0.102 | 0.102 | 0.102 | 0.102 | 0.102 | 0.102 | 1.0 |
| **In vivo fu,p=LF/LT0** | -- | 0.102 | 0.102 | 0.070 | 0.206 | 0.206 | 0.206 | 0.102 | 0.102 | 0.102 | 1.0 |
| **Capillary KG=k1/k2** | uM | 3.3 | 3.3 | 19 | 19 | 19 | 19 | 3.3 | 3.3 | 3.3 | 3.3 |
| **Capillary KA=k7/k8** | uM | 290 | 290 | 290 | 290 | 290 | 290 | 290 | 290 | 290 | 290 |
| **Tissue KP=k6/k5** | nM | 87 | 87 | 87 | 8.7 | 8.7 | 870 | 87 | 87 | 87 | 87 |

| **Supplementary Table S2.**  **Brain imipramine (Mathematica Solve-steady state model)**  *Yellow highlight indicates change in parameter relative to simulation 1 (11)* | | | | | | | | | | | | | |
| --- | --- | --- | --- | --- | --- | --- | --- | --- | --- | --- | --- | --- | --- |
| Parameter/Variable | units | Simulation number (simulation number in Table IV) | | | | | | | | | | | |
|  |  | 1 (11) | 2 (12) | 3 (13) | 4 (14) | 5 (15) | 6 (19) | 7 (16) | 8 (17) | 9 (18) | 10 (20) | 11 | 12 |
| **k1** | min^-1^ | 5,400 | 72 | 5,400 | 54,000 | 540 | 5,400 | 72 | 5,400 | 5,400 | 5,400 | 72 | 72 |
| **k2** | nM^-1^min^-1^ | 0.06 | 0.06 | 0.06 | 0.6 | 0.006 | 0.06 | 0.06 | 0.06 | 0.06 | 0.06 | 0.06 | 0.06 |
| **k3** | min^-1^ | 150 | 150 | 150 | 150 | 150 | 150 | 150,000 | 150 | 150 | 150 | 150,000 | 150 |
| **k4** | min^-1^ | 2.1 | 2.1 | 2.1 | 2.1 | 2.1 | 2.1 | 2,100 | 21 | 2.1 | 21 | 2,100 | 2.1 |
| **k5** | nM^-1^min^-1^ | 0.006 | 0.006 | 0.006 | 0.006 | 0.006 | 0.006 | 0.006 | 0.006 | 0.006 | 0.006 | 0.006 | 0.006 |
| **k6** | min^-1^ | 0.5 | 0.5 | 0.5 | 0.5 | 0.5 | 0.5 | 0.5 | 0.5 | 0.5 | 0.5 | 0.5 | 0.5 |
| **k7** | min^-1^ | 6000 | 252 | 6000 | 6000 | 6000 | 6000 | 252 | 6000 | 6000 | 6000 | 252 | 252 |
| **k8** | nM^-1^min^-1^ | 0.006 | 0.006 | 0.006 | 0.006 | 0.006 | 0.006 | 0.006 | 0.006 | 0.006 | 0.006 | 0.006 | 0.006 |
| **k9** | min^-1^ | 0 | 0 | 0 | 0 | 0 | 6 | 0 | 0 | 6 | 6 | 6 | 6 |
| **k10** | min^-1^ | 60 | 60 | 60 | 60 | 60 | 6 | 60 | 60 | 60 | 60 | 60 | 60 |
| **KG (p11)** | nM | 1,200 | 1,200 | 1,200 | 1,200 | 1,200 | 1,200 | 1,200 | 1,200 | 1,200 | 1,200 | 1,200 | 1,200 |
| **KA (p12)** | nM | 42,000 | 42,000 | 42,000 | 42,000 | 42,000 | 42,000 | 42,000 | 42,000 | 42,000 | 42,000 | 42,000 | 42,000 |
| **GT0 (p13)** | nM | 20,000 | 20,000 | 70,000 | 20,000 | 20,000 | 20,000 | 20,000 | 20,000 | 20,000 | 20,000 | 20,000 | 20,000 |
| **AF (p14)** | nM | 800,000 | 800,000 | 600,000 | 800,000 | 800,000 | 800,000 | 800,000 | 800,000 | 800,000 | 800,000 | 800,000 | 800,000 |
| **PT (p15)** | nM | 5,000 | 5,000 | 5,000 | 5,000 | 5,000 | 5,000 | 5,000 | 5,000 | 5,000 | 5,000 | 5,000 | 5,000 |
| **LT0 (p16)** | nM | 100 | 100 | 100 | 100 | 100 | 100 | 100 | 100 | 100 | 100 | 100 | 100 |
| **VP (p17)** | L/kg | 0.01 | 0.01 | 0.01 | 0.01 | 0.01 | 0.01 | 0.01 | 0.01 | 0.01 | 0.01 | 0.01 | 0.01 |
| **VT (p18)** | L/kg | 0.7 | 0.7 | 0.7 | 0.7 | 0.7 | 0.7 | 0.7 | 0.7 | 0.7 | 0.7 | 0.7 | 0.7 |
| **GL0** | nM | 45.3 | 45.3 | 79.2 | 45.3 | 45.3 | 45.3 | 45.3 | 45.3 | 45.3 | 45.3 | 45.3 | 45.3 |
| **AL0** | nM | 51.9 | 51.9 | 19.4 | 51.9 | 51.9 | 51.9 | 51.9 | 51.9 | 51.9 | 51.9 | 51.9 | 51.9 |
| **LF0** | nM | 2.73 | 2.73 | 1.36 | 2.73 | 2.73 | 2.73 | 2.73 | 2.73 | 2.73 | 2.73 | 2.73 | 2.73 |
| **GL** | nM | 11.3 | 45.3 | 33.1 | 11.0 | 14.1 | 1.13 | 45.3 | 11.3 | 6.13 | 8.97 | 39.9 | 43.6 |
| **AL** | nM | 39.5 | 51.9 | 25.1 | 39.6 | 38.3 | 3.94 | 51.9 | 39.5 | 20.8 | 31.1 | 42.8 | 49.1 |
| **LF** | nM | 49.2 | 2.73 | 41.9 | 49.4 | 47.6 | 4.86 | 2.73 | 49.2 | 25.6 | 38.6 | 2.13 | 2.54 |
| **GF** | nM | 19,988 | 19,954 | 69,966 | 19,989 | 19,985 | 19,998 | 19,955 | 19,988 | 19,944 | 19,941 | 19,960 | 19,956 |
| **LM** | nM | 50.2 | 2.78 | 42.7 | 50.3 | 48.6 | 1.29 | 2.78 | 5.02 | 6.78 | 3.06 | 2.16 | 0.672 |
| **PL** | nM | 1,880 | 162 | 1,695 | 1,884 | 1,843 | 76 | 161 | 284 | 376 | 177 | 126 | 40 |
| **PF** | nM | 3,120 | 4,838 | 3,305 | 3,116 | 3,157 | 4,924 | 4,838 | 4,716 | 4,624 | 4,823 | 4,873 | 4,960 |
| **LM+PL** | nM | 1,930 | 165 | 1,738 | 1,934 | 1,892 | 77 | 164 | 289 | 383 | 180 | 128 | 41 |
| **Kp,brain=(LM+PL)/LT0** | -- | 19.3 | 1.6 | 17.4 | 19.3 | 18.9 | 0.77 | 1.6 | 2.9 | 3.8 | 1.8 | 1.3 | 0.41 |
| **In vitro fu,p=LF0/LT0** | -- | 0.027 | 0.027 | 0.014 | 0.027 | 0.027 | 0.027 | 0.027 | 0.027 | 0.027 | 0.027 | 0.027 | 0.027 |
| **In vivo fu,p=LF/LT0** | -- | 0.492 | 0.027 | 0.419 | 0.494 | 0.476 | 0.049 | 0.027 | 0.492 | 0.256 | 0.386 | 0.021 | 0.025 |
| **Capillary KG=k1/k2** | uM | 90 | 1.2 | 90 | 90 | 90 | 90 | 1.2 | 90 | 90 | 90 | 1.2 | 1.2 |
| **Capillary KA=k7/k8** | uM | 1,000 | 42 | 1,000 | 1,000 | 1,000 | 1,000 | 42 | 1,000 | 1,000 | 1,000 | 42 | 42 |
| **Tissue KP=k6/k5** | nM | 83 | 83 | 83 | 83 | 83 | 83 | 83 | 83 | 83 | 83 | 83 | 83 |
